# *Rhodococcus rhodochrous* IEGM 1362 Immobilized in Macroporous PVA Cryogel as an Effective Biocatalyst for the Production of Bioactive (–)-Isopulegol Compounds

**DOI:** 10.3390/ph18060839

**Published:** 2025-06-03

**Authors:** Polina Y. Maltseva, Natalia A. Plotnitskaya, Alexandra A. Chudinova, Irina V. Ilyina, Konstantin P. Volcho, Nariman F. Salakhutdinov, Irina B. Ivshina

**Affiliations:** 1 Institute of Ecology and Genetics of Microorganisms, Perm Federal Research Center of the Ural Branch of the Russian Academy of Sciences, 13 Golev Str., 614081 Perm, Russia; inbox.98@bk.ru (P.Y.M.); luchnikova.n@mail.ru (N.A.P.); 2 Department of Microbiology and Immunology, Perm State University, 15 Bukirev Str., 614990 Perm, Russia; 79194890159@yandex.ru; 3 N.N. Vorozhtsov Novosibirsk Institute of Organic Chemistry of the Siberian Branch of the Russian Academy of Sciences, 9 Lavrentiev Avenue, 630090 Novosibirsk, Russia; ilyina@nioch.nsc.ru (I.V.I.); volcho@nioch.nsc.ru (K.P.V.); anvar@nioch.nsc.ru (N.F.S.)

**Keywords:** biotransformation, (–)-isopulegol, immobilization, polyvinyl alcohol, *Rhodococcus*

## Abstract

**Background**: This study explored the biotransformation of (–)-isopulegol using immobilized cells of *Rhodococcus rhodochrous* IEGM 1362 to optimize the production of new bioactive compounds. **Methods**: An efficient biocatalyst based on *R*. *rhodochrous* IEGM 1362 cells immobilized in a macroporous polyvinyl alcohol (PVA) cryogel matrix was developed for the production of bioactive derivatives of (–)-isopulegol. The biological characteristics of the immobilized cells were investigated using scanning and transmission electron microscopy and energy-dispersive X-ray spectroscopy methods. **Results**: The use of the biocatalyst increased the overall yield of target products from 54% with free cells to 87% with immobilized cells in a single cycle. Major derivatives identified included (1*R*,2*S*,5*R*)-5-(hydroxymethyl)-2-(prop-1-en-2-yl)cyclohexanol and (1*R*,3*R*,4*S*)-3-hydroxy-4-(prop-1-en-2-yl)cyclohexanecarboxylic acid, both exhibiting potential pharmacological activity. The biocatalyst retained functional activity toward monoterpenoid over 13 exploitation cycles, meeting industrial biotechnology requirements. Immobilized cells were characterized by the absence of endogenous reserve inclusions (in particular lipids) and a high intracellular iron content. **Conclusions**: The developed immobilized biocatalyst is promising for scaling up the production of biologically active compounds.

## 1. Introduction

(–)-Isopulegol (C_10_H_18_O, CAS 89-79-2) is a monoterpene alcohol widely used as a source material for the synthesis of biologically active compounds due to its low cost and high availability [1]. (–)-Isopulegol-based compounds possessing antiviral [2], analgesic [3], antioxidant, antimicrobial [4], and antiproliferative [5] activities have been obtained using chemical synthesis methods. Currently available information is limited to fragmentary reports on (–)-isopulegol conversion by cutinases from *Aspergillus oryzae* and *Humicola insolens* [6], along with its whole-cell degradation by *Rhodococcus* sp. [7]. However, the scientific literature lacks data on either the specific metabolites generated through microbial transformation or their biotechnological potential. In this context, actinomycetes of the genus *Rhodococcus* are of particular interest as they demonstrate a pronounced catalytic activity in the transformation of complex hydrophobic compounds [8].

**Figure 1:**
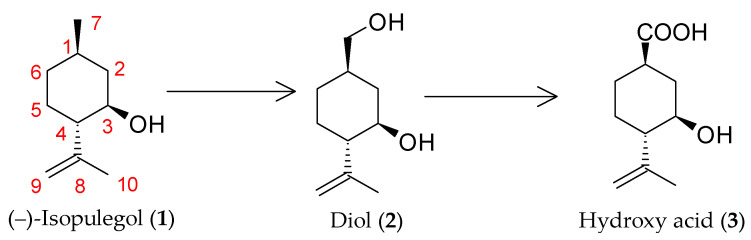
Biotransformation of (–)-isopulegol by *R*. *rhodochrous* IEGM 1362 free cells [9].

We previously [9] selected the *R*. *rhodochrous* strain IEGM 1362, which is capable of transforming (–)-isopulegol into Me-7 oxidation products—(1*R*,2*S*,5*R*)-5-(hydroxymethyl)-2-(prop-1-en-2-yl)cyclohexanol (diol **2**) and (1*R*,3*R*,4*S*)-3-hydroxy-4-(prop-1-en-2-yl)cyclohexanecarboxylic acid (hydroxy acid **3**) (Figure 1). Antitumor and analeptic activities were predicted for both compounds. However, the overall yield of the target products was 25% (9.34% for diol **2** and 15.38% for hydroxy acid **3**), representing a relatively low value and indicating the need for process optimization.

One common approach to optimizing the microbial transformation of complex organic compounds is the immobilization of bacterial cells on the surface or within the matrix of a carrier. Cell immobilization enhances stability, increases resistance to environmental factors (e.g., temperature, pH), and improves productivity and reusability, thereby eliminating the need for regular cultivation of the target strain [10,11,12].

Currently, a wide range of biocatalytic systems based on synthetic polymers have been developed and are used for the synthesis of antibiotics, enzymes, and the precursors of biologically active compounds [12]. The use of synthetic carriers—particularly polyvinyl alcohol (PVA) cryogel—for immobilization has several significant advantages. The macroporous structure of PVA cryogranules facilitates the mass transfer of substrate to microbial cells and enables the transport of transformation products into the culture medium [13]. PVA is characterized by high mechanical strength and resistance, non-toxicity toward living organisms, as well as availability and low cost. Additionally, the immobilized biocatalyst can retain metabolic activity even after long-term storage [14].

Among alternative immobilization matrices, alginate gels are widely used due to their mild gelation conditions and biocompatibility [15]. However, alginate matrices exhibit low mechanical stability, which limits their application in prolonged biotransformations. Silica-based carriers, such as mesoporous silica and silica gels, provide a high surface area and thermal stability but require complex chemical modification for immobilizing living cells [16]. These factors support the use of PVA as a matrix for the immobilization of *Rhodococcus* spp. cells to obtain pharmaceutically valuable derivatives of (–)-isopulegol.

The aim of this study was to optimize the biotransformation of (–)-isopulegol using *R*. *rhodochrous* IEGM 1362 cells immobilized in macroporous PVA cryogel cells in order to produce new bioactive compounds and pharmaceutical ingredients.

## 2. Results and Discussion

### 2.1. Biotransformation of (–)-Isopulegol Using Immobilized Cells

The immobilization of *R*. *rhodochrous* IEGM 1362 cells in PVA granules preserved their functional activity toward (–)-isopulegol over 13 cycles of exploitation, indicating the potential of the obtained biocatalyst to optimize and scale up the process of monoterpenoid biotransformation to obtain pharmacologically valuable derivatives.

During the initial cycles of using inoculated PVA granules, the active growth and adaptation of the immobilized cells to the cultivation conditions—particularly to the presence of a substrate—likely occurred, leading to the achievement of maximum catalytic activity.

**Figure 2:**
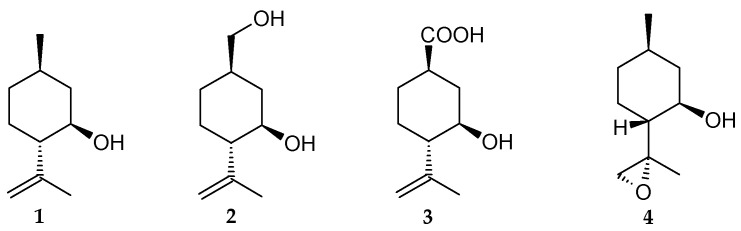
Chemical structures of (–)-isopulegol (**1**) and its transformation products (**2**–**4**) by immobilized *R*. *rhodochrous* IEGM 1362.

According to gas chromatography–mass spectrometry (GC-MS) data, the main products of the biotransformation of (–)-isopulegol (**1**) by immobilized *R*. *rhodochrous* IEGM 1362 cells are diol (**2**), hydroxy acid (**3**), and (*S*)-isopulegol epoxide (**4**), with the preferential formation of hydroxy acid (Figure 2). A typical chromatogram of the biotransformation products is shown in Figure S1 in Supplementary Materials.

As shown previously, compounds **2** and **3** may exhibit anticancer activity and are promising as respiratory analeptics (Table 1) [9]. According to Paulino et al. [17] the presence of hydroxyl groups in the chemical structure of monoterpenoids enhances antimicrobial activity, suggesting the potential use of the obtained metabolites as antibacterial agents.

By-product **4**, identified as (*S*)-isopulegol epoxide, is a commercially available compound. Based on *in silico* analysis, this compound does not demonstrate significant potential biological activity (Table 1).

**Figure 3:**
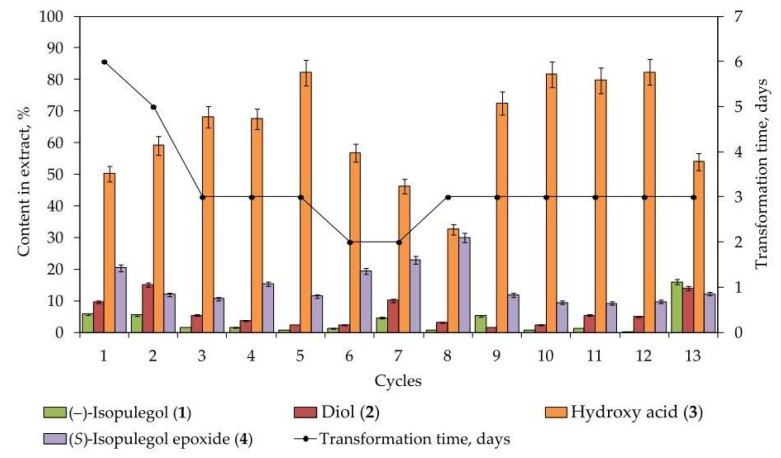
Changes in ethyl acetate extract (%) composition during biotransformation of (–)-isopulegol by immobilized *R*. *rhodochrous* IEGM 1362.

The selectivity of hydroxy acid (**3**) formation increases from cycle 1 to cycle 5, then decreases, reaching a minimum in cycle 8. It subsequently rises again to 72.5–82.4% in the following cycles (from 9 to 12) (Figure 3). The pronounced decrease in selectivity during cycle 8 appears to be due to the enhanced formation of (*S*)-isopulegol epoxide (**4**), as well as to the accumulation of other by-products (*m*/*z* 156 and 182). The recovery of high selectivity in cycles 9–12 suggests possible catabolic stabilization as the cells adapt to changing conditions.

Based on the residual content of the initial (–)-isopulegol in the reaction mixtures, it can be concluded that the activity of cells immobilized in PVA granules remained high throughout the first 12 cycles and decreased during the 13th cycle. Further process optimization should focus on selecting appropriate cultivation parameters to suppress epoxide formation and minimize the yield of by-products.

### 2.2. Morphology and Elemental Composition of R. rhodochrous IEGM 1362 Immobilized Cells

To assess the micromorphological characteristics of free and immobilized bacterial cells, electron microscopy methods were employed. For scanning electron microscopy (SEM) and transmission electron microscopy (TEM), samples of PVA granules with immobilized *R*. *rhodochrous* IEGM 1362 cells were prepared in their initial stage and after eight cycles of continuous biocatalyst use.

**Figure 4:**
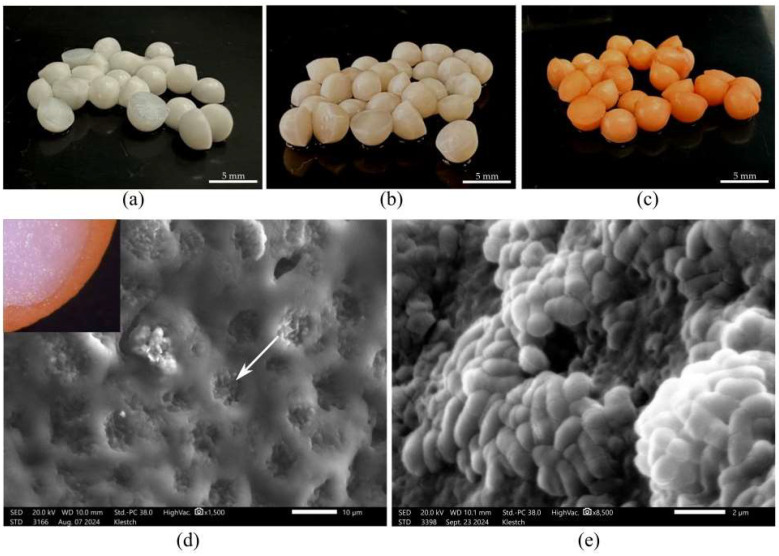
The appearance of the granules (**a**–**c**) and SEM (**d**,**e**). (**a**) Sterile granules after 8 cycles with (–)-isopulegol; (**b**) inoculated granules after 1 cycle with (–)-isopulegol; (**c**) inoculated granules after 8 cycles with (–)-isopulegol; and (**d**,**e**) the surface of inoculated granules after 8 cycles with (–)-isopulegol. The arrow indicates the cells’ aggregates in the macropores of PVA.

As shown in Figure 4a–c, by the 8th cycle, the inoculated granules had the pronounced orange pigmentation characteristic of *R*. *rhodochrous* colonies, indicating the colonization of the carrier by rhodococci. The SEM and TEM of the inoculated PVA granules revealed clusters of *R*. *rhodochrous* IEGM 1362 cells located within the pores of the cryogel, formed during the freeze–thaw process (Figure 4d, indicated by the arrow), as well as the presence of isolated, compact multicellular aggregates (Figure 4e).

**Figure 5:**
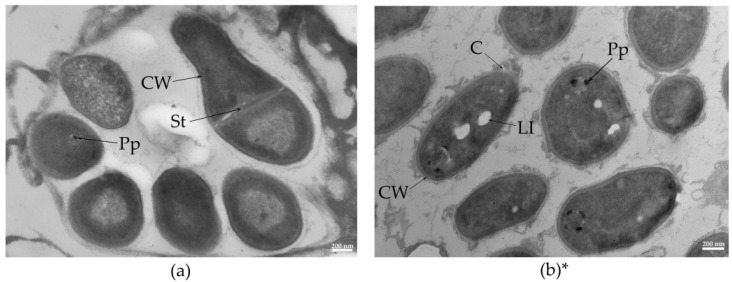
The TEM of *R*. *rhodochrous* IEGM 1362 cells immobilized in PVA after 8 cycles with (–)-isopulegol (**a**) and free *R*. *rhodochrous* IEGM 1362 cells (**b**). Designations: C, capsular layer; CW, cell wall; Pp, polyphosphate particles; LI, lipid inclusions; St, cell division septa. * Modified from [9].

In the ultrathin sections of rhodococci included in a PVA matrix, no intracellular lipid inclusions were detected (Figure 5a). This suggests that the carrier protects the cells from adverse environmental conditions, thereby reducing the cells’ need for the additional storage of endogenous reserves.

Free cells were characterized by a differentiated external capsule layer in the form of specific surface protrusions (Figure 5b). Since the cell wall plays a key role in the interaction between bacteria and the substrate, it can be assumed that the increased cell surface area, resulting from hypertrophic cell wall growth, serves to form a kind of reservoir that facilitates the fixation of the substrate and its subsequent oxidation.

In contrast, cells immobilized in the PVA matrix do not require such an adaptation, as the structure of the carrier itself promotes substrate accumulation within macropores, enabling more efficient access and utilization by the bacterial cells.

**Figure 6:**
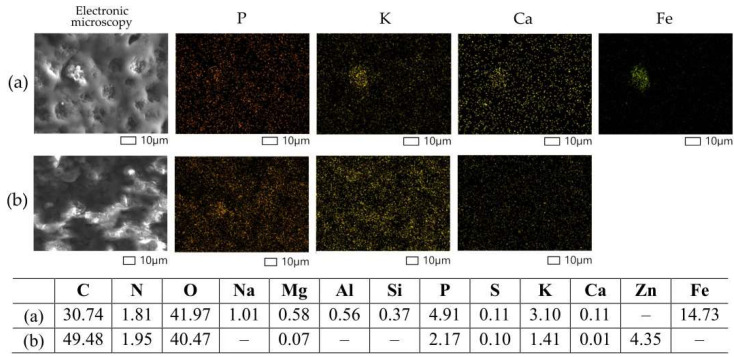
Elemental mapping and composition (mass %) of *R*. *rhodochrous* IEGM 1362 cells immobilized in PVA after 8 cycles with **(a**) and without (**b**) (–)-isopulegol determined using X-ray microanalysis. Distribution of individual chemical elements is highlighted with color.

Immobilized cells used over eight cycles of (–)-isopulegol biotransformation exhibited a pronounced increase in magnesium, phosphorus, potassium, and calcium content (Figure 6a), along with a greater diversity of detected elements, including sodium, aluminum, silicon, and iron (Figure 6a).

Iron is known to play a key role in cellular respiration and metabolism and is an essential component of redox partners for many enzymes, significantly influencing bacterial catalytic activity [18]. For example, oxygenases of the cytochrome P450 family, involved in the transformations of monoterpenoids by rhodococci [19,20,21,22], contain an iron-porphyrin complex in their structure. Moreover, negatively charged bacterial cells can attract metal cations, whose neutralization increases the hydrophobicity of the cell surface, thereby enhancing the cells’ ability to adsorb hydrophobic organic compounds [23]. According to Liu et al. [24], the presence of iron ions in the culture medium of *Rhodococcus* induces the activation of transport systems responsible for the uptake of polycyclic aromatic hydrocarbons, particularly pyrene, increasing its biodegradation rate [24]. In our study, iron likely contributes to the enhanced biocatalytic activity of immobilized cells through various mechanisms, including the activation of oxidative enzymes and the modification of the cell surface.

### 2.3. Viability of Immobilized R. rhodochrous IEGM 1362 Cells

One of the key operational characteristics of biocatalysts is the guaranteed preservation of the viability of immobilized bacterial cells. Determining the number of viable cells is associated with several challenges, as immobilized microorganisms are firmly embedded in the carrier matrix and cannot be extracted without compromising cell integrity. Consequently, the use of direct methods for counting viable cells is not feasible. Alternative methods include assessing respiratory activity and employing Live/Dead fluorescent staining.

**Figure 7:**
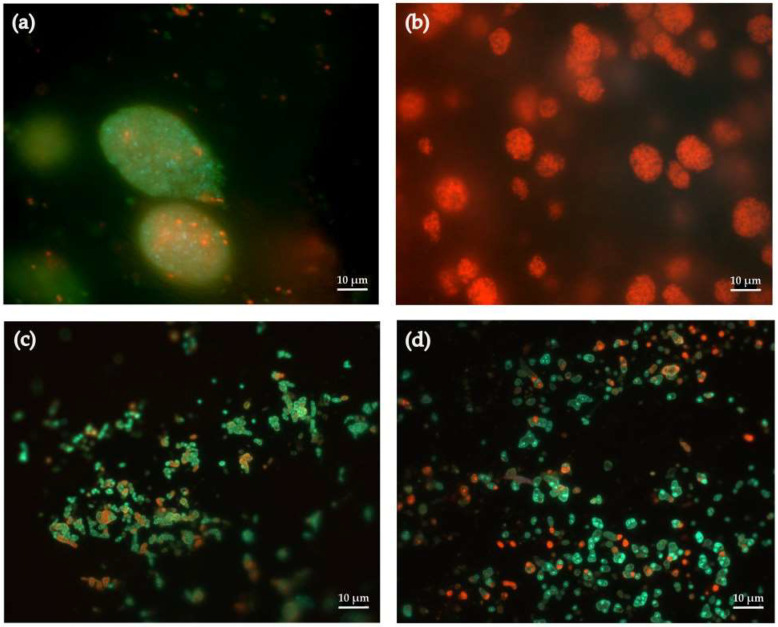
Fluorescent microscopy (1000×) of immobilized *R*. *rhodochrous* IEGM 1362 cells after 1 (**a**,**c**) and 13 (**b**,**d**) cycles with (–)-isopulegol stained with Live/Dead: (**a**,**b**) granule outer surface; (**c**,**d**) the thickness of the granule. Green fluorescence indicates living cells, red fluorescence indicates dead cells.

Using the dye, we found that by the 13th cycle of (–)-isopulegol biotransformation, the surface pores of the granules contained non-viable cells of *R*. *rhodochrous* IEGM 1362, whereas during cycles 1–12, actively metabolizing cells predominated in the same regions (Figure 7a,b). The observed “sealing” of surface pores (Figure 7b) correlates with a decrease in the system’s biocatalytic activity, as confirmed by GC-MS data (see Figure 3). This phenomenon is likely related to the exhaustion of the carrier’s pore capacity while nutrient diffusion is still maintained. As a result, necrotic bacterial biomass accumulates progressively, forming a mechanical barrier that may contribute to the blockage of substrate transport [25,26].

It is worth noting that in the deeper layers of the carrier, the ratio of live and dead cells remained stable even after multiple cycles of biocatalyst use (Figure 7c,d), indicating heterogeneity in the bacterial population, similar to the structure of biofilms. Surface zones are more vulnerable to environmental stress, whereas internal zones provide more stable conditions that support long-term cell viability.

The findings above demonstrate the potential for further optimization of the carrier architecture to minimize nutrient gradients and extend the number of exploitation cycles of the immobilized biocatalyst.

**Figure 8:**
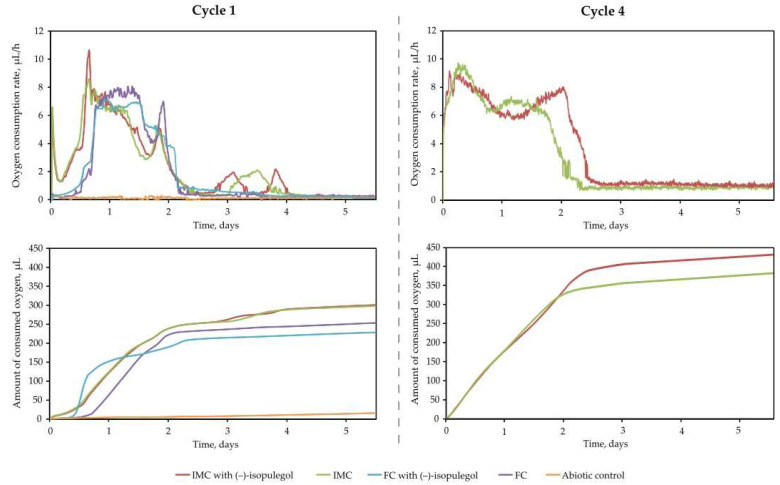
The rate of oxygen consumption and the amount of oxygen consumed by cells immobilized in PVA (IMCs) and free (FCs) *R*. *rhodochrous* IEGM 1362 cells during the 1st and 4th cycles of (–)-isopulegol biotransformation.

Analysis of the respiratory activity of rhodococci showed that the immobilized *R*. *rhodochrous* IEGM 1362 cells demonstrated a reducted lag phase during the first cycle of exploitation compared to free cells, both with and without the addition of (–)-isopulegol (Figure 8).

As the number of biotransformation cycles increased, the lag phase completely disappeared, and the stationary phase became more prolonged. This was reflected in the graphs of the rate and total volume of oxygen consumed during the fourth cycle. The recorded increase in total oxygen consumption (on 30%) during this period indicated the enhanced metabolic activity of the immobilized cells.

The extended duration of the stationary phase—during which the maximum yield of target products was observed—may be associated with enhanced cellular adaptation to the specified conditions and, consequently, increased stress resistance due to immobilization within the macroporous PVA cryogel matrix.

It is known that the immobilization of bacterial cells leads to significant changes in their biochemical profile, similar to those observed in biofilm-associated cells under natural conditions. Immobilized cells exhibit a pronounced increase in the concentration of key biomolecules, including polysaccharides, compared to free cells, with differences potentially reaching several-fold [27]. In immobilized systems, polysaccharides serve as a structural component and an adhesion factor, although to a lesser extent than in biofilms [28].

**Figure 9:**
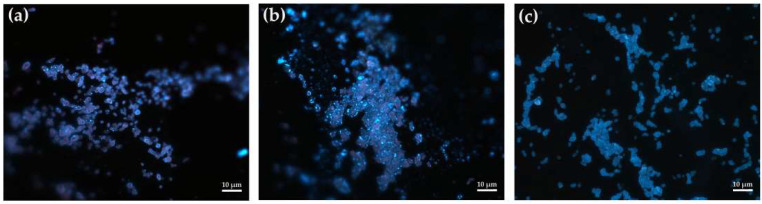
The fluorescent microscopy (1000×) of immobilized (**a**,**b**) and free (**c**) *R*. *rhodochrous* IEGM 1362 cells stained with the Calcofluor White stain after the 1st (**a**) and 4th (**b**) cycles of (–)-isopulegol biotransformation.

To study the adaptive responses of immobilized *R. rhodochrous* IEGM 1362 cells—including extracellular polymer synthesis and quorum-sensing mechanisms—we performed staining with Calcofluor White, a fluorescent dye specific to β-polysaccharides. Microscopic analysis of the stained sections of the inoculated granules revealed polysaccharide structures in the form of discrete droplets localized on the surfaces of bacterial cells (Figure 9). This distribution pattern contrasts with that observed in biofilms, where polysaccharides typically form a continuous mucous matrix covering cell aggregates.

As shown in Figure 9, after four cycles of exploitation, there is a noticeable increase in the number of immobilized cells secreting exopolysaccharides. This may be due to the cells’ adaptation to repeated use, providing protection against stresses associated with the biotransformation of (–)-isopulegol. Furthermore, the accumulation of exopolysaccharides may contribute to the enhanced stability and prolonged viability of immobilized cells during extended operational periods.

## 3. Materials and Methods

### 3.1. Bacterial Strain

*R*. *rhodochrous* IEGM 1362 was isolated from the Paltinskoye peat deposit, Perm region, Russia, and deposited in the Regional Specialized Collection of Alkanotrophic Microorganisms (acronym IEGM, WFCC number 285). The strain not only transforms (–)-isopulegol but also utilizes *n*-hexadecane as a sole carbon source, degrades dehydroabietic acid, exhibits resistance to Cr^6+^ (40.0 mM), Mo^6+^, and Zn^2+^ (5.0 mM), and transforms the monoterpenoids (–)-cis-carveol, (–)-trans-carveol, and (–)-L-carvone (http://www.iegmcol.ru/strains/rhodoc/rhodoch/r_rhod1362.html, accessed on 27 April 2025).

### 3.2. Chemicals

All commercially available chemicals and solvents were of reagent quality and utilized without additional treatment unless stated otherwise. (–)-Isopulegol (purity ≥ 98%, MW 154.25 g/mol, C_10_H_18_O, (1*R*,2*S*,5*R*)-5-methyl-2-(prop-1-en-2-yl)cyclohexan-1-ol, CAS No. 89-79-2, ${\left[\alpha \right]}_{D}^{31}$ = −21 (c = 0.4, CHCl_3_)) was obtained from Sigma-Aldrich (St. Louis, MO, USA). Polyvinyl alcohol mark 16/1 GOST 10779-78 was purchased from “Nevinnomysskiy azot” (Nevinnomyssk, Russia).

### 3.3. Immobilization of Bacterial Cells

For immobilization, bacterial cells were initially cultured in nutrient broth (Sigma-Aldrich, St. Louis, MO, USA) at 28 °C and 160 rpm for 48 h. The resulting cell suspension (OD_600_ 1.6, (8.9 ± 0.7) × 10^8^ CFU/mL) in phosphate buffer (pH 7.0) was mixed with a sterile 12% PVA solution at a 1:2 (*v*/*v*) ratio. The mixture was aliquoted in 96-well microplates and subjected to three freeze–thaw cycles (at −18 °C and 4 °C, respectively) [29]. Before use, the inoculated cryogranules were rehydrated in 0.5% NaCl solution for 24 h.

### 3.4. Biotransformation of (–)-Isopulegol

The biotransformation process was carried out in 100 mL Erlenmeyer flasks containing 20 mL of RS mineral salt medium (g/L: K_2_HPO_4_—2.0; KH_2_PO_4_—2.0; KNO_3_—1.0; (NH_4_)_2_SO_4_—2.0; NaCl—1.0; MgSO_4_—0.2; CaCl_2_—0.02, FeCl_3_ × 7H_2_O—0.001), under shaking conditions (160 rpm) at 28 °C. For each biotransformation experiment, 40 PVA cryogranules were added to 20 mL of RS medium. The cultivation medium was supplemented with yeast extract (0.1 g/L), and trace element solution prepared according to Postgate (0.1% *v*/*v*). (–)-Isopulegol (Sigma-Aldrich, St. Louis, MO, USA) was added at a final concentration of 0.025% *v*/*v*. After each biotransformation cycle, the granules were washed from the medium and rinsed with phosphate buffer (pH 7.0).

The controls were: (1) immobilized cells in a mineral medium without (–)-isopulegol (biotic control); (2) sterile PVA cryogranules in a mineral medium with (–)-isopulegol (abiotic control); (3) bacterial suspension in a mineral medium with (–)-isopulegol (control of transformation by free cells).

### 3.5. Analytical Methods

To isolate the residual (–)-isopulegol and its derivatives, the post-culture medium was acidified with a 10% HCl solution, followed by the addition of an equal volume of ethyl acetate. After the separation of the emulsion, the organic phase was collected. For further extraction, the remaining aqueous phase was subjected to two additional extractions with ethyl acetate to ensure maximum recovery. The combined ethyl acetate extracts were concentrated using a rotary evaporator Laborota 4000 (Heidolph, Schwabach, Germany). The resulting concentrate was then sequentially washed with a 1% aqueous NaHCO_3_ solution and distilled water until a neutral pH (~7.0) was achieved, then dried over anhydrous Na_2_SO_4_. The purified residue obtained was subsequently used for analytical procedures.

Qualitative analysis was conducted via thin-layer chromatography (TLC) on Alugram^®^Xtra SIL G/UV254 plates (Macherey-Nagel, Düren, Germany). Chromatographic separation employed a mobile phase consisting of *n*-hexane and ethyl acetate mixed in equal volumes (1:1, *v*/*v*). After development, the plates were air-dried and subsequently exposed to iodine vapor to visualize the separated compounds.

The biotransformation products of (–)-isopulegol were analyzed using GC–MS data. To identify isopulegol’s biotransformation products, compounds **2** and **3** obtained according to [9] and epoxide **4** synthesized according to [30] were used. Analyses were performed using an Agilent 7890A gas chromatograph (Agilent Technologies, Santa Clara, CA, USA) coupled with an Agilent 5975C quadrupole mass spectrometer detector (Agilent Technologies). Separation was achieved on an HP-5MS quartz capillary column measuring 30 m in length and 0.25 mm in internal diameter. He (1 atm) served as the carrier gas. The column temperature was programmed to increase from 50 to 280 °C. Mass spectra were acquired over a mass-to-charge (*m*/*z*) range of 50 to 700 and subsequently matched against reference spectra from the NIST08 database for compound identification.

### 3.6. Scanning Electron Microscopy (SEM), Transmission Electron Microscopy (TEM), and Energy-Dispersive X-Ray Spectroscopy (EDX) with Elemental Mapping

For SEM, fragments of inoculated PVA granules were fixed with 1.5% (*w*/*v*) glutaraldehyde and washed, dropped onto coverslips, and air-dried. The coverslips were then mounted on stubs and coated with Au in a JFC-1100 ion sputter (Jeol, Tokyo, Japan). Imaging was performed using a JSM-IT200 electron microscope (Jeol, Tokyo, Japan).

For TEM, thin sections were prepared using an 8800 Ultrotome III (LKB-Produkter, Stockholm, Sweden) and stained with lead citrate. Ultrathin sections were examined using a JEM-1400 electron microscope (Jeol, Tokyo, Japan). TEM-EDX analysis with elemental mapping was performed on the same JEM-1400 microscope (Jeol, Tokyo, Japan) equipped with an energy-dispersive X-ray analysis system (EDXA, Inca Energy-350, Oxford Instruments, Abingdon, UK), operating at an accelerating voltage of 80 keV (tilt angle, 15°). EDX spectra and elemental maps were obtained using AZtec software (Oxford Instruments, Abingdon, UK).

SEM, TEM, and EDX analysis were performed at the Core Facility Center “UNIQEM Collection” at the Research Center of Biotechnology, Russian Academy of Sciences.

### 3.7. Respiratory Activity

The oxygen consumption rates and total oxygen uptake by *Rhodococcus* cells were measured using a 10-channel respirometer Columbus Micro-Oxymax (Columbus Instruments, Columbus, OH, USA). Experiments were conducted in 300 mL flasks containing 100 mL RS medium with stirring (300 rpm, 28 ± 2 °C) on an Ikamag^®^ RO10 power magnetic stirrer (IKA-Werke, Staufen, Germany).

### 3.8. Fluorescent Microscopy

The granule sections were visualized using an optical microscope Axio Imager M2 (Carl Zeiss Microscopy GmbH, Jena, Germany) with an Axiocam 506 Color camera and a fluorescent light source HXP 120 V (Zeiss, Germany) in fluorescence mode (light filter FS 106). PVA granules were stained with the Live/Dead^®^ Bac*Light*^TM^ Bacterial Viability Kit (Invitrogen, Carlsbad, CA, USA) to differentiate living and dead cells, and with Calcofluor White Stain (Sigma-Aldrich, Darmstadt, Germany) to detect β-polysaccharides. 

### 3.9. In Silico Analysis of (–)-Isopulegol and Its Derivatives

The predicted biological activities of the (–)-isopulegol derivatives were accessed using the PASS software (Prediction of Activity Spectra for Substances, http://www.pharmaexpert.ru/passonline/index.php, accessed on 27 April 2025). This computational tool estimates the biological potential of compounds based on their molecular structures by comparing them with a large database of known biologically active substances. The output includes a spectrum of possible biological activities along with their associated probabilities of occurrence (P_a_). The highest P_a_ value for biological activity was taken as 1.

## 4. Conclusions

The obtained results indicate the feasibility of using *Rhodococcus rhodochrous* IEGM 1362 cells immobilized in a macroporous polyvinyl alcohol (PVA) cryogel matrix for the efficient production of bioactive monoterpenoid (–)-isopulegol derivatives. The biocatalyst retains operational activity over 13 cycles and increases the overall yield of the target products from 54 to 87% per cycle compared to the use of free cells.

The biological features of the immobilized cells, such as the absence of intracellular lipid inclusions and high iron content, were investigated. A promising direction for further research involves optimizing this biocatalytic process and integrating immobilized cell systems into continuous reactors to scale up the synthesis of pharmaceutically important monoterpenoid compounds. Additional perspectives include detailed investigation of the metabolic pathways and regulatory mechanisms underlying biocatalyst activity to reduce unwanted by-products. Finally, expanding the application of this immobilized cell system to synthesize a broader range of pharmaceutically relevant monoterpenoid derivatives represents a valuable avenue for future research.

## Figures and Tables

**Table 1:** Estimated biological activity of (–)-isopulegol and its derivatives *.

Estimated Activity	(–)-Isopulegol (1)	Diol (2)	Hydroxy Acid (3)	(*S*)-Isopulegol Epoxide (4)
Carminative	0.976	0.930	0.928	0.835
Anti–eczematic	0.929	0.918	0.908	0.844
Neuromuscular acetyl choline blocker	0.751	0.677	0.682	–
Inhibitor of β-glucuronidase	–	0.670	0.714	0.329
Immunosuppressive	0.755	0.720	0.731	0.631
Stimulator of NFκB transcription factor	0.747	0.730	0.730	–
Inhibitor of retinol dehydrogenase	0.738	0.739	0.693	0.277
Respiratory analeptic	–	0.568	0.686	0.588
Anti-inflammatory	0.690	0.645	0.692	0.413
Antitumor	–	0.550	0.482	–

* Modified from [9].

## Data Availability

The data presented in this study are available in the Supplementary Materials or can be obtained on request from the corresponding author.

## Supplementary Materials

The following supporting information can be downloaded at: https://www.mdpi.com/article/10.3390/ph18060839/s1, Figure S1: Typical chromatogram of biotransformation of products of (–)-isopulegol by immobilized cells of *Rhodococcus rhodochrous* IEGM 1362.

## Abbreviations

The following abbreviations are used in this manuscript:

PVA	Polyvinyl alcohol
SEM	Scanning electron microscopy
TEM	Transmission electron microscopy
EDX	Energy dispersive X-ray spectroscopy
GC-MS	Gas chromatography–mass spectrometry
