# Supplementary material for: Rhodococcus rhodochrous IEGM 1362 Immobilized in Macroporous PVA Cryogel as an Effective Biocatalyst for the Production of Bioactive (–)-Isopulegol Compounds
Source: Pharmaceuticals (Basel). 2025 Jun 3;18(6):839. doi: 10.3390/ph18060839 (PMC12195689; doi:10.3390/ph18060839)
Supplement: Supplementary file 1 [file pharmaceuticals-18-00839-s001.zip › pharmaceuticals-3658153-supplementary.pdf]

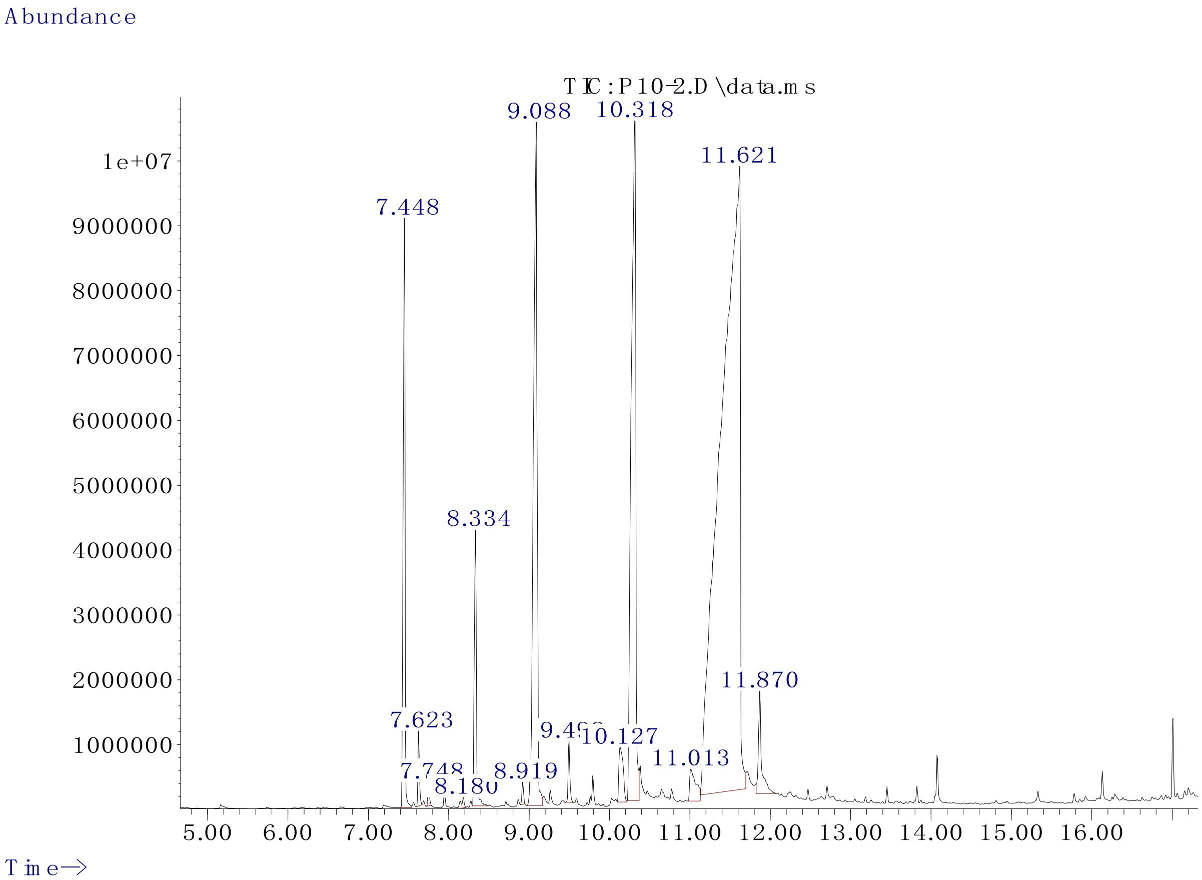

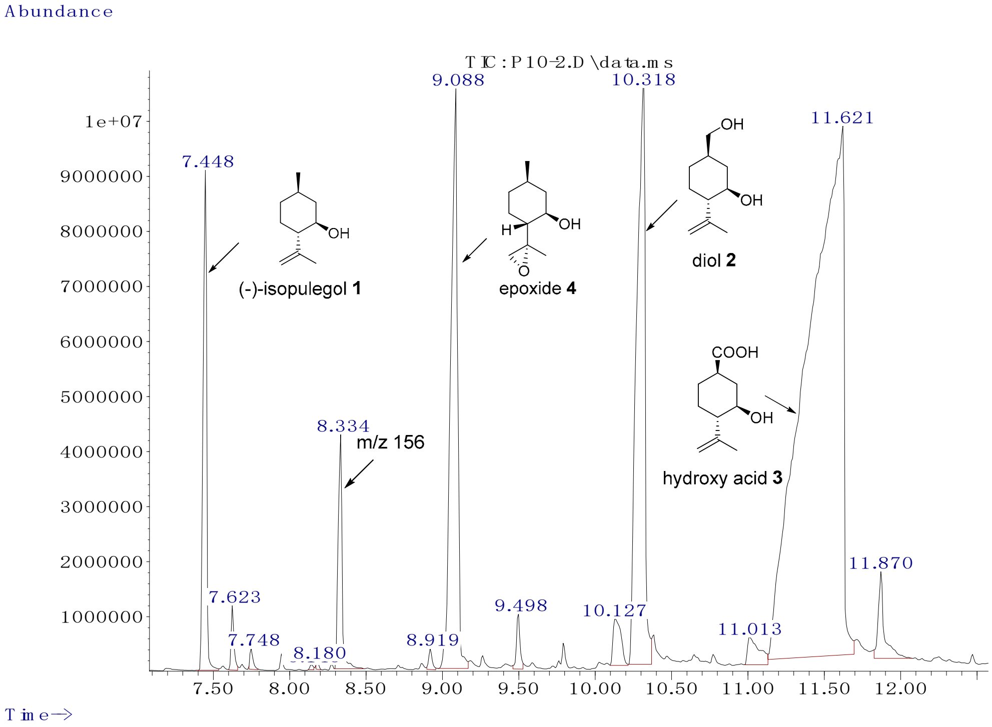

Figure S1. Typical chromatogram of products of (–)-isopulegol biotransformation by immobilized cells of *Rhodococcus rhodochrous* IEGM 1362
